# DFT Meets Wave-Function Methods for Accurate Structures and Rotational Constants of Histidine, Tryptophan, and Proline

**DOI:** 10.1021/acs.jpca.3c04227

**Published:** 2023-09-04

**Authors:** Vincenzo Barone, Lina Marcela Uribe Grajales, Silvia Di Grande, Federico Lazzari, Marco Mendolicchio

**Affiliations:** † Scuola Normale Superiore di Pisa, Piazza dei Cavalieri 7, 56126 Pisa, Italy; ‡ Scuola Superiore Meridionale, Largo San Marcellino 10, 80138 Napoli, Italy

## Abstract

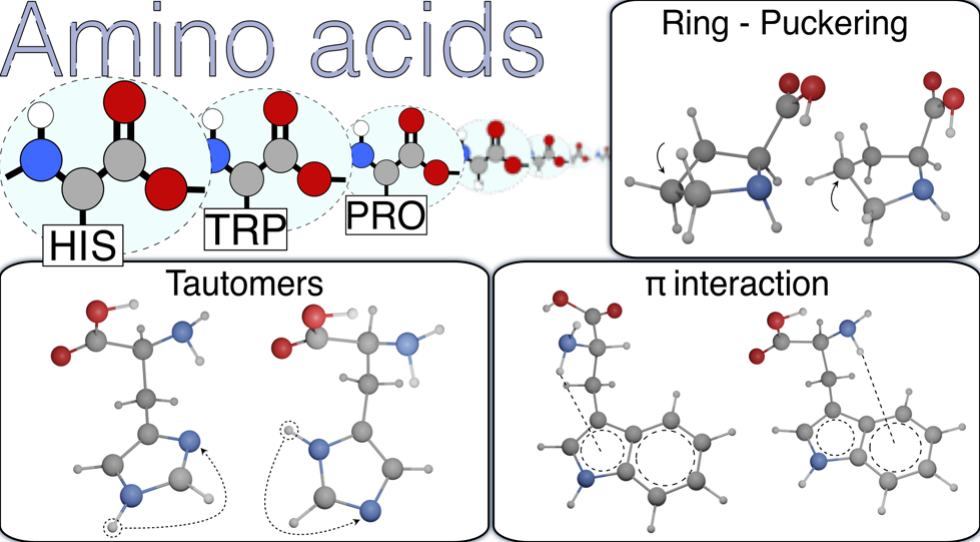

A new computational strategy has been applied to the conformational and spectroscopic properties in the gas phase of amino acids with very distinctive features, ranging from different tautomeric forms (histidine) to ring puckering (proline), and heteroaromatic structures with non-equivalent rings (tryptophan). The integration of modern double-hybrid functionals and wave-function composite methods has allowed us to obtain accurate results for a large panel of conformers with reasonable computer times. The remarkable agreement between computations and microwave experiments allows an unbiased interpretation of the latter in terms of stereoelectronic effects.

## Introduction

Increasing attention has been paid in the last years to the conformational landscape of amino acids, which couple limited dimensions with a remarkable flexibility tuned by different kinds of non-covalent interactions.^1,2^ Since environmental effects can strongly modify the characteristics of amino acids (for instance, zwitterionic forms are more stable in crystals^3^ and aqueous solutions,^4^ whereas neutral forms are exclusively found in the gas phase^5^ or in inert matrixes^6^), an unbiased disentanglement of intrinsic stereoelectronic features requires preliminary studies in the gas phase. Thanks to the development of spectrometers coupling supersonic-jet expansion^7^ and laser ablation,^8^ thermolabile molecules with high melting points (like most amino acids) have become accessible to high-resolution spectroscopy studies. However, the interpretation of experimental spectra in structural and thermochemical terms is made difficult by the fast relaxation of some conformers to more stable counterparts whenever the corresponding energy barriers can be overcome under the specific experimental conditions.^9−11^ Quantum chemical (QC) computations can help solve this kind of problems, provided that they couple accuracy^12−14^ and feasibility for large numbers of different structures of medium-sized molecules.^15−17^ Furthermore, an effective exploration of flat potential energy surfaces (PESs) requires more refined strategies^18,19^ with respect to the systematic searches and/or local optimization techniques routinely employed for small semirigid molecules. In our opinion, the most suitable approach involves the synergistic use of QC methods of increasing sophistication in the different steps of an exploration/exploitation workflow driven by machine learning (ML) tools.^20−22^ In this framework, after the preliminary discovery of conformers lying in a sufficiently large energy range by relatively cheap methods, the structures of the most stable conformers are refined by a double-hybrid functional and, possibly, further improved by a linear regression approach (LRA) involving a few empirical parameters in order to correct systematic errors.^23,24^ Next, the transition states (TSs) ruling interconversion paths between pairs of conformers are found and energy minima connected to more stable species by low energy barriers are removed from the conformer list. Improved relative energies of the surviving conformers are evaluated by single-point computations with a wave-function composite method^25−30^ and used, together with zero-point energies (ZPEs), computed by the double-hybrid functional mentioned above, to determine the final relative populations. Finally, spectroscopic parameters of the energy minima with non-negligible populations are computed.^16,31^

Systematic studies based on this strategy have confirmed that the backbone of natural amino acids containing simple non-polar side chains shows two main hydrogen bond patterns (usually referred to as type I and type II).^5,32−34^ On the other hand, polar side chains give access to backbone–(side chain) hydrogen bonds, with this strongly increasing the number of low-energy conformers.^35−39^ Additional interactions between backbone polar hydrogen atoms and side chain π-systems are possible for amino acids containing aromatic moieties.^40−42^ This diversified landscape can be further enriched by a comprehensive analysis of amino acids showing additional features, like tautomerism (histidine, His), ring puckering (proline, Pro), or heteroaromatic structures with non-equivalent rings (tryptophan, Trp). Since all of these amino acids have been investigated in the gas phase by microwave (MW) spectroscopy,^38,43−45^ the QC results must match those accurate experimental data. In this framework, the most distinctive feature of the present analysis with respect to previous studies is the coupling of feasibility and accuracy (relative mean unsigned errors (RMUEs) within 0.3% for rotational constants and 1% for quadrupolar coupling constants and relative energies), allowing the *a priori* prediction of experimental outcomes without any *ad hoc* assumption.

## Methods

As already mentioned in the Introduction, a preliminary exploration of the conformational PES by a fast semiempirical method^46^ guided by ML algorithms^22,47^ is followed by a characterization of low-energy conformers at the B3LYP-D3BJ/6-31+G* level.^48,49^ The same combination of functional and basis set (hereafter B3/SVP) will be used also for the computation of anharmonic contributions (vide infra). The geometries of conformers lying within 1500 cm^–1^ above the absolute energy minimum are refined by the revDSD-PBEP86-D3BJ double-hybrid functional^50−53^ (hereafter rDSD) in conjunction with the jun-cc-pVTZ basis set^52^ (hereafter j3). While the rDSD/j3 model provides excellent conformational landscapes^15,54,55^ and geometrical parameters,^24^ further refinements are needed for structures lying below 1000 cm^–1^ and not connected to more stable energy minima by barriers lower than 400 cm^–1^.^56,57^ In fact, the main outcomes of experimental MW spectra are the ground-state rotational constants (*B*_τ_^0^, where τ refers to the inertial axes *a*, *b*, *c*), which include, together with equilibrium rotational constants (*B*_τ_^eq^), also electronic contributions (neglected in the following due to their very small values) and vibrational corrections (*ΔB*_τ_^vib^).^16,58,59^ The leading (and most expensive) contribution to vibrational corrections comes from cubic force constants.^60^ Fortunately, these terms can be obtained at affordable levels of theory (B3/SVP in the present context) since errors of the order of 10% on vibrational corrections correspond to errors lower than 0.1% on the overall rotational constants.^16,61^ On the other hand, errors in the same range for equilibrium rotational constants can be obtained only by state-of-the-art QC methods.^62−65^ As already mentioned in the Introduction, the systematic nature of the errors allows significant improvement of the rDSD/j3 results by the LRA;^24,66,67^ however, the use of empirical parameters is not fully satisfactory. Therefore, an extensive benchmark of different basis sets and additional contributions was performed, which led to the selection of the cc-pVTZ-F12 basis set^68^ (hereafter 3F12) and to the inclusion of core–valence (CV) correlation at the MP2 level in conjunction with the cc-pwCVTZ^69^ (hereafter wC3) basis set. These choices define the new Pisa composite scheme (PCS),^70^ in which each geometrical parameter (*r*) is obtained by combining the corresponding parameters optimized at different levels
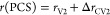
1where *r*_V2_ is the geometrical parameter computed including an estimate of valence correlation energy with methods not exceeding the MP2 level (rDSD/3F12 in the present case) and *Δr*_CV2_ is the CV correction obtained from the difference between all-electron (ae) and frozen core (fc) MP2 computations in conjunction with the wC3 basis set. Several test computations have shown that PCS geometrical parameters are extremely accurate, and this will be further checked in the present context with reference to low-lying conformers of His, Trp, and Pro.

Since all amino acids contain at least one nitrogen atom, ^14^N nuclear quadrupole coupling constants (χ_*ii*_, with *i* referring to the inertia axis *a*, *b*, or *c*) play a non-negligible role in the accurate predictions of rotational spectra.^62,71^ Furthermore, the intensities of the different MW transitions are determined by the components of dipole moments (μ_*i*_).^58,71^ While both dipole moments and quadrupole coupling constants can be computed with sufficient accuracy at the rDSD level,^39,72^ accurate relative energies determining the conformer populations can be obtained by single-point energy evaluations on top of PCS geometries using composite wave-function methods rooted in the coupled cluster (CC) ansatz including single, double, and (perturbatively) triple excitations CCSD(T).^73,74^ The final expression of the PCS energy is analogous to that of the PCS geometrical parameters, but now *E*_V2_ includes the CBS extrapolation and a further term (*ΔE*_V_) is added to take into account valence correlation beyond the MP2 level. The CBS extrapolations appearing in both the *E*_V2_ and *ΔE*_V_ terms are performed by the standard *n*^–3^ two-point formula.^75^ In order to allow the inclusion of those contributions, the *E*_V2_ term is evaluated at the MP2 level in place of the rDSD level employed for geometries
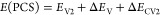
2where
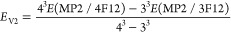
3and
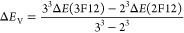
4with
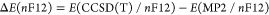
5Finally
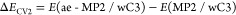
6

In the equations above, all the energies are obtained within the fc approximation, unless the label ae (all-electron) is explicitly employed.

Finally, the ZPEs required for the computation of standard enthalpies at 0 K (Δ*H*_0_^°^) are evaluated in the framework of vibrational perturbation theory to second order (VPT2),^76−78^ employing rDSD/3F12 harmonic frequencies^31^ and B3/SVP anharmonic contributions,^79,80^ except in the case of tryptophan, where also harmonic contributions have been obtained at the B3/SVP level.

The aim of the PCS model is to approach the accuracy of CCSD(T)+CBS+CV computations at the cost of a triple-ζ CCSD(T) computation and without any empirical parameter thanks to the evaluation of CBS and CV contributions by the inexpensive MP2 model. While use of smaller basis sets requires the introduction of empirical factors,^81^ the success of the “cheap” family of methods^26^ witnesses that this goal can be reached starting from triple-ζ basis sets, and the PCS model further improves the results. Furthermore, single-point CCSD(T) energy evaluations with triple-ζ basis sets are feasible today for very large molecules thanks to the implementation of linear-scaling algorithms possibly employing local-correlation treatments.^82−84^ The situation is different for gradient evaluations, where fast implementations are available only for DFT and MP2 (hence double-hybrids), which are, therefore, employed in the PCS model. In any case, since the dimensions of the studied molecules are small enough to allow the use of conventional approaches, all the computations have been performed with the Gaussian package.^85^

## Results and Discussion

The B3/SVP equilibrium rotational constants and vibrational corrections of all of the molecules studied in the present work are collected in Table 1. It is apparent that, as mentioned in the Introduction, vibrational corrections are of the order of 1% of the corresponding rotational constants. As a consequence, they cannot be neglected when aiming at unbiased comparisons with experiments.

**Table 1:** Equilibrium Rotational Constants and Vibrational Corrections for the Species Detected in MW Experiments Computed at the B3/SVP Level^a^

	*B*_*a*_^eq^	*B*_*b*_^eq^	*B*_*c*_^eq^	*ΔB*_*a*_^vib^	*ΔB*_*b*_^vib^	*ΔB*_*c*_^vib^
Imidazole	9698	9363	4764	–79.9	–77.2	–41.0
Indole	3872	1629	1146	–29.9	–10.0	–7.3
Histidine ϵIIgg^–^	1838	822	740	–13.9	–2.6	–2.6
Tryptophan IIgg	1235	391	346	–11.3	–1.6	–1.5
Tryptophan IIg^–^g	1293	332	287	–9.5	–2.5	–1.9
Proline IE^–^ b	3926	1541	1341	–44.3	–16.5	–14.7
Proline IE^+^ b	3995	1551	1269	–44.6	–13.6	–12.9
Proline IIE^–^	3717	1651	1382	–46.9	–7.1	–6.0
Proline IIE^+^ b	3981	1570	1256	–46.6	–13.6	–9.7

a All of the values are given in MHz.

b The lowest frequency normal mode has been left harmonic.

Before analyzing the specific targets of this work, let us consider the semirigid molecules corresponding to the side chains of histidine and tryptophan, namely, imidazole and indole. Since MW spectra are available for both molecules, a first estimate of the reliability of different methods can be obtained in the absence of the additional challenges related to backbone flexibility. The maximum and mean unsigned errors (MAX and MUE, respectively) are used together with their relative values (RMAX and RMUE) to analyze the quality of the results delivered by different computational models.

The results collected in Table 2 confirm that at most qualitative trends can be obtained by the methods usually employed for the interpretation of MW spectra (B3LYP and MP2), whereas the LRA based on rDSD/j3 computations confirms its remarkable performance. However, the new PCS approach delivers comparable results without the need for any empirical parameter besides those already present in the underlying electronic structure method. At this level, both CV correlation and vibrational corrections need to be included since they play an opposite role, but the issuing error compensation is far from being perfect.

**Table 2:** Comparison between the Experimental and Computed Rotational Constants of Imidazole and Indole^a^

Molecule	Parameter	Exp.^b^	rDSD/j3	LRA^c^	PCS^c^	B3/j3	MP2/j3
imidazole	*B*_*a*_	9725.3	9755.6	9721.5	9723.7	9756.4	9755.9
	*B*_*b*_	9374.0	9406.7	9370.3	9380.7	9463.6	9403.5
	*B*_*c*_	4771.9	4789.0	4769.6	4772.8	4803.9	4788.2
	MAX		32.7	3.8	6.7	32.0	30.6
	MUE		26.7	3.3	3.1	24.5	25.5
	RMAX		0.36%	0.05%	0.07%	0.67%	0.34%
	RMUE		0.34%	0.04%	0.03%	0.37%	0.32%
indole	*B*_*a*_	3877.8	3891.0	3876.2	3880.0	3907.0	3885.6
	*B*_*b*_	1636.0	1638.9	1635.5	1636.7	1642.9	1642.7
	*B*_*c*_	1150.9	1153.2	1150.5	1151.4	1156.6	1154.6
	MAX		13.2	1.6	2.2	29.2	7.8
	MUE		6.1	0.8	1.1	13.9	6.1
	RMAX		0.34%	0.04%	0.06%	0.75%	0.41%
	RMUE		0.24%	0.03%	0.05%	0.56%	0.31%

a All the values (except relative errors) are in MHz.

b From ref (86) for imidazole and ref (87) for indole.

c Includes B3/SVP vibrational corrections from Table 1.

**Figure 1:**
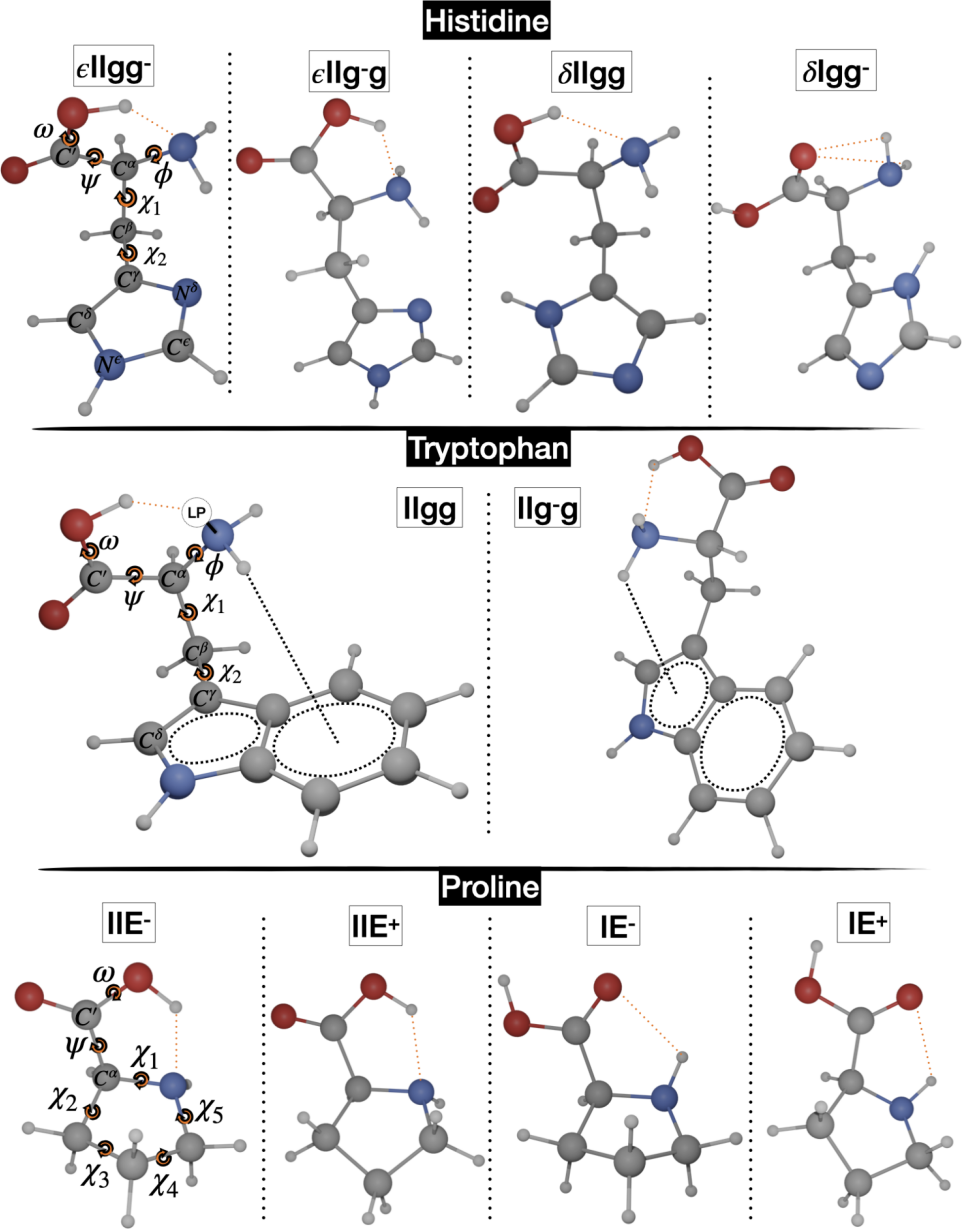
Low-energy minima of histidine, tryptophan, and proline.

The “soft” dihedral angles governing the conformational landscape of His and Trp belong either to the backbone (ϕ = LP–N–C^α^–C′ and ψ = N–C^α^–C′–O(H) dihedral angles) or to the side chain (χ_1_ = N–C^α^–C^β^–C^γ^ and χ_2_ = C^α^–C^β^–C^γ^–N^δ^ for histidine or χ_2_ = C^α^–C^β^–C^γ^–C^δ^ for tryptophan). LP is the nitrogen lone-pair perpendicular to the plane defined by the two amine hydrogens and the C^α^ atom (Figure 1). Only nearly planar conformations are allowed for the carboxy moiety of all amino acids (ω = C^α^–C′–O–H ≈ 0° or 180°), with ω ≈ 0° being preferred, unless the oxidryl hydrogen is involved in strong hydrogen bonds with other electronegative atoms. The *c*, *g*, *s*, and *t* labels are then used to indicate the cis, gauche, skew, and trans conformations determined by the “soft” dihedral angles in the following order: ϕ, ψ, χ_1_, and χ_2_. In the case of Pro, the ψ and ω dihedral angles retain the same definitions, but the puckering of the pyrrolidine ring must be properly defined.^88^ A full description of five-membered rings requires in principle two pseudorotation coordinates, the puckering amplitude (α) and the phase angle (τ),^89,90^ which can be obtained from the endocyclic torsion angles χ_1_, ..., χ_5_ (see Figure 1):
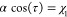
7
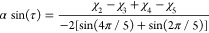
8Two tautomeric forms are possible for histidine, depending on the presence of an acidic hydrogen on N^δ^ or N^ϵ^ (referred to as δ and ϵ in the following).

In agreement with previous computational studies,^91^ the exploration of the conformational landscape of His provided several low-energy structures, most of which are stabilized by hydrogen bonds of type I (bifurcated NH_2_···O=C, ϕ ≈ 180°, ψ ≈ 180°, ω ≈ 180°, *ttt*) and II (N···HO, ϕ ≈ 0°, ψ ≈ 0°, ω ≈ 0°, *ccc*).^8^

As already reported for other amino acids^39^ some low-energy conformers of type III (ϕ ≈ 180°, ψ ≈ 0°, ω ≈ 180°, *tct*) have been found, but they can easily relax to more stable I conformers overcoming the very small energy barriers governing rotation around the ψ dihedral angle. Also the number of detectable conformers of type I is reduced by fast relaxations through nearly free rotation around the χ_1_ dihedral angle. Therefore, our computations suggest that only four conformers might be detected in MW experiments. The corresponding structures are shown in Figure 1, and the main structural and energetic features are collected in Table 3 and Table S1 of the Supporting Information. Contrary to the usual situation for aliphatic amino acids,^39^ conformers of type II are more stable than their type I counterparts (in spite of a less favorable orientation of the OH group in the carboxy moiety) since only the former conformers can establish a favorable interaction between the aromatic π-system of imidazole and one aminic hydrogen of the backbone.^92^ The different intramolecular hydrogen bonding networks ruling the relative stability of type II structures have been recently analyzed,^91^ and our results confirm the main conclusion of that work. The results collected in Table 3 show that ZPEs tune the relative stability of the type I and type II conformers.

**Table 3:** rDSD/3F12 Relative Electronic Energies (Δ*E*_rDSD_) and Harmonic Zero-Point Energies (ΔZPE_H_), Together with Differences between PCS and rDSD/3F12 Electronic Energies (ΔPCS) and B3/SVP Anharmonic Corrections to ZPEs (ΔZPE_A–H_) for the Low-Lying Tautomers and Conformers of Histidine^a^

Label	Δ*E*_rDSD_	ΔPCS	ΔZPE_H_^b^	ΔZPE_(A–H)_^c^	Δ*H*_0_^°^ d	ϕ	ψ	ω	χ_1_	χ_2_
ϵIIgg^–^	0	0	0	0	0	–8	9	–2	59	–72
ϵIIg^–^g	395	18	5 (15)	31	449	13	–15	3	–67	65
δIIgg	165	–6	32 (37)	95	286	–17	20	–6	59	75
δIgg^–^	1005	–10	–128 (−120)	88	955	82	–174	179	67	–49

a Best estimates of relative enthalpies at 0 K (Δ*H*_0_^°^) and dihedral angles (Figure 1) optimized at the PCS level are also given. The angles are in degrees, whereas all of the energetic quantities are in cm^–1^.

b At the rDSD/3F12 level and (in parentheses) at the B3/SVP level.

c At the B3/SVP level.

d Sum of columns 2, 3, 4, and 5.

Furthermore, the difference between rDSD/3F12 and PCS relative electronic energies does not exceed 20 cm^–1^ and that between B3/SVP and rDSD/3F12 relative ZPEs does not exceed 10 cm^–1^. The former results confirm the reliability of the rDSD/3F12 model, whereas the second result permits confident use of B3/SVP ZPEs for larger molecules, like tryptophan.

Despite the accessible relative energy of the species δIIgg and ϵIIg^–^g (δIIa and ϵIIb according to the nomenclature of ref (45)) only the most stable species ϵIIgg^–^ (ϵIIa according to the nomenclature of ref (45)) has been detected in the experimental MW study.^45^ The PCS spectroscopic parameters are given in Table 4 together with their experimental counterparts. Comparison of Table 4 with the results of previous investigations^45^ shows that our computational approach reduces the RMUE on rotational constants by about 1 order of magnitude (0.27% at the PCS level and 2.32% from the MP2 computations reported in ref (45)), which, in absolute terms, translates into a PCS maximum error of about 4 MHz (to be compared to 27 MHz at the MP2 level).

**Table 4:** Ground-State Rotational Constants (*B*_*a*_^0^, *B*_*b*_^0^, and *B*_*c*_^0^ in MHz) and ^14^N-Nuclear Quadrupole Coupling Constants (χ in MHz) of the ϵIIgg^–^ Structure (ϵIIa according to the Nomenclature of ref (45)) of Histidine

Parameter	Experiment^a^	PCS^b^	MP2^a^
*B*_*a*_^0^	1847.5	1844	1839
*B*_*b*_^0^	831.7	834	859
*B*_*c*_^0^	745.9	748	770
χ_*aa*_/N^δ^	1.611	1.63	1.62
χ_*bb*_/N^δ^	–3.497	–3.61	–3.49
χ_*cc*_/N^δ^	1.886	1.97	1.87
χ_*aa*_/N^ϵ^	–0.179	–0.18	–0.18
χ_*bb*_/N^ϵ^	1.122	1.12	0.97
χ_*cc*_/N^ϵ^	–0.943	–0.93	–0.79
χ_*aa*_/N^*a*^	0.005	–0.02	0.04
χ_*bb*_/N^*a*^	2.098	2.31	2.10
χ_*cc*_/N^*a*^	–2.103	–2.29	–2.14

a From ref (45).

b PCS equilibrium geometries, rDSD/3F12 properties, and B3/SVP vibrational corrections from Table 1.

This finding shows that PCS computations are accurate enough to assign the detected conformer on the basis of rotational constants since the differences between the computed values of different structures (see Table 1 in ref (45)) are larger than the maximum error. As already mentioned, the nitrogen atoms of His (N^*a*^ in the amino group of the backbone and N^δ^, N^ϵ^ in the imidazole ring of the side chain; see Figure 1) give rise to quadrupole coupling constants. The results collected in Table 4 show that the experimental and computed values are in remarkable agreement. It is also noteworthy that the PCS value of the HNC^α^C′ dihedral angle (−21.8°) is very close to that estimated in ref (45) in order to minimize the difference between computed and experimental quadrupole coupling constants.

In agreement with previous studies,^38,93,94^ the backbone of the most stable Trp conformers shows a hydrogen bond pattern of type II. Furthermore, the preferred conformation of the χ_2_ dihedral angle (governing the position of the indole ring) is close to 90° (broadly referred to as *g*) and the most stable structures are characterized by the interaction of one aminic hydrogen of the backbone with the π-system of the phenyl (χ_1_ ≈ 60°) or pyrrole (χ_1_ ≈ −60°) ring of indole. The structures of the two most stable conformers (IIgg and IIg^–^g or IIb+ and IIc+ according to the nomenclature of ref (38)) are shown in Figure 1, while their main features are collected in Table 5 and Table S2 of the Supporting Information. It is quite apparent that the stabilizing effect of interactions involving the phenyl ring (IIgg conformer) is considerably stronger, with respect to those in which the pyrrole ring is engaged (IIg^–^g conformer). In analogy with the case of histidine, a single conformer (IIgg) was initially detected in MW studies. However, analysis of other isotopologues pointed out the presence of a second less abundant species. In fact, both ^14^N and ^15^N isotopologues could be observed for the nitrogen atoms of Trp (N^*a*^ in the amino group and N^ϵ^ in the pyrrole ring; see Figure 1). Since our computations should be sufficiently accurate to discriminate between isotopologues, we compare in Table 6 the computed and experimental values. The agreement between theory and experiment is indeed remarkable, and the errors are once again about an order of magnitude smaller than those delivered by previous computations. As a consequence, the computed rotational constants allow the unbiased assignment of the detected species, with quadrupolar coupling constants further confirming the results.

**Table 5:** rDSD/3F12 Relative Electronic Energies (Δ*E*_rDSD_), Together with Differences between PCS and rDSD/3F12 Electronic Energies (ΔPCS), B3/SVP Harmonic Zero-Point Energies (ΔZPE_H_), and Anharmonic Corrections to ZPEs (ΔZPE_A–H_) for the Low-Energy Conformers of Tryptophan^a^

Label	Δ*E*_rDSD_	ΔPCS	ΔZPE_H_^b^	ΔZPE_(A–H)_^b^	Δ*H*_0_^°^ c	ϕ	ψ	ω	χ_1_	χ_2_
IIgg	0	0	0	0	0	–12	14	–3	56	84
IIg^–^g	353	37	–21	–7	362	15	–17	4	–62	109

a Best estimates of relative enthalpies at 0 K (Δ*H*_0_^°^) and dihedral angles (see Figure 1) optimized at the PCS level are also given. The angles are in degrees, whereas all the energetic quantities are in cm^–1^.

b At the B3/SVP level.

c Sum of columns 2, 3, 4, and 5.

**Table 6:** Experimental Ground-State Rotational Constants (*B*_*a*_^0^, *B*_*b*_^0^, and *B*_*c*_^0^ in MHz) and ^14^N-Nuclear Quadrupole Coupling Constants (χ in MHz) of the Two Most Stable Tryptophan Conformers Compared with Computed Values^a^

	IIgg						IIg^–^g			
	^14^N_*i*_–^14^N_*a*_		^15^N_*i*_–^14^N_*a*_		^15^N_*i*_–^15^N_*a*_		^15^N_*i*_–^14^N_*a*_		^15^N_*i*_–^15^N_*a*_	
Param.	Exp.^b^	Calc.^c^	Exp.^b^	Calc.^c^	Exp.^b^	Calc.^c^	Exp.^b^	Calc.^c^	Exp.^b^	Calc.^c^
*B*_*a*_^0^	1243.6	1237	1231.1	1225	1219.5	1213	1281.3	1286	1272.5	1273
*B*_*b*_^0^	392.5	395	392.2	394	391.3	394	333.7	344	332.4	333
*B*_*c*_^0^	346.9	349	345.7	348	344.3	346	287.1	288	286.3	287
χ_*aa*_/N^*a*^	0.31^d^	–0.1					–2.33	–2.34		
χ_*bb*_/N^*a*^	1.71	2.2					1.95	2.16		
χ_*cc*_/N^*a*^	–2.02	–2.1					0.38	0.18		
χ_*aa*_/N^ϵ^	1.08	1.0								
χ_*bb*_/N^ϵ^	1.30	1.4								
χ_*cc*_/N^ϵ^	–2.38	–2.4								

a Rotational constants of the ^15^N isotopomers are also reported.

b From ref (38).

c PCS equilibrium geometries, rDSD/3F12 properties, and B3/SVP vibrational corrections from Table 1.

d Fixed in the fitting.

Proline is the only natural amino acid whose side chain closes a (pyrrolidinic) cycle. Exhaustive conformational searches produced six low-energy conformers with two representatives each for the I, II, and III forms. However, the two species of type III are too unstable (more than 1000 cm^–1^ above the absolute energy minimum) to be detected in MW studies. The other four low-energy species adopt an envelope (E) arrangement with either *exo*- or *endo*-like placements of the carboxy moiety. The puckering amplitude α is always very close to 40°, and the phase angle τ is close either to 90° (E^+^, *exo* COOH) or −90° (E^–^, *endo* COOH)^88,95^ (see Figure 1). All of those species have actually been detected in MW experiments,^43,44^ and their structural and energetic parameters are given in Table 7 and Table S3 of the Supporting Information.

**Table 7:** rDSD/3F12 Relative Electronic Energies (Δ*E*_rDSD_) and Harmonic Zero-Point Energies (ΔZPE_H_), Together with Differences between PCS and rDSD/3F12 Electronic Energies (ΔPCS) and B3/SVP Anharmonic Corrections to ZPEs (ΔZPE_A–H_) for the Low-Energy Structures of Proline^a^

Label	Δ*E*_rDSD_	ΔPCS	ΔZPE_H_^b^	ΔZPE_(A–H)_^c^	Δ*H*_0_^°^ d	ψ	ω	τ
IIE^–^	0	0	0	0	0	2	–1	–90
IIE^+^	217	–17	–15 (−14)	9	194	1	2	88
IE^–^	625	–64	–105 (−126)	9	465	169	179	–88
IE^+^	605	–25	–139 (−157)	12	453	174	179	102

a Best estimates of relative enthalpies at 0 K (Δ*H*_0_^°^) and dihedral angles (see Figure 1) optimized at the PCS level are also given. The angles are in degrees, whereas all the energetic quantities are in cm^–1^.

b At the rDSD/3F12 level and (in parentheses) at the B3/SVP level.

c At the B3/SVP level.

d Sum of columns 2, 3, 4, and 5.

It is apparent that species of type II are significantly more stable than their counterparts of type I and that *exo* or *endo* placements of the carboxyl groups have comparable energies. The relative stabilities of the different species obtained at the rDSD/3F12 level are in fair agreement with their PCS counterparts (MAX = 64 cm^–1^ and MUE = 35 cm^–1^), which are, in turn, close (MAX = 28 cm^–1^ and MUE = 18 cm^–1^) to the values obtained in ref (88) employing the so-called focal point analysis (FPA). Actually, the difference between rDSD/3F12 and PCS relative energies is often of the same order of magnitude as the corresponding difference between B3/SVP and rDSD/3F12 harmonic ZPEs or between harmonic and anharmonic ZPEs. This finding confirms that none of these contributions can be neglected in order to reach fully converged values.^96^

The experimental spectroscopic parameters are compared with the computed parameters in Table 8. It is quite apparent that the presence of large-amplitude puckering of the pyrrolidine ring increases the errors of the computed rotational constants with respect to those obtained for the other amino acids, with the effect being particularly strong for the *B*_*a*_ rotational constant of the IE^+^ species. However, even in those circumstances, the agreement between computed and experimental rotational constants remains sufficiently good to permit the unequivocal assignment of the detected species, which is, anyway, further confirmed by quadrupolar coupling constants.

**Table 8:** Experimental Ground-State Rotational Constants (*B*_*a*_^0^, *B*_*b*_^0^, and *B*_*c*_^0^ in MHz) and ^14^N-Nuclear Quadrupole Coupling Constants (χ in MHz) of the Four Most Stable Proline Species Compared with Computed Values

	IE^–^		IE^+^		IIE^–^		IIE^+^	
Param.	Exp.^a^	Calc.^b^	Exp.^a^	Calc.^b^	Exp.^a^	Calc.^b^	Exp.^a^	Calc.^b^
*B*_*a*_^0^	3857.2	3879	4004.0	4017	3673.9	3684	3923.6	3966
*B*_*b*_^0^	1590.5	1573	1567.3	1556	1688.4	1682	1605.9	1583
*B*_*c*_^0^	1377.5	1365	1281.5	1272	1407.4	1405	1279.8	1266
χ_*aa*_/N	2.47	2.71	1.14	1.24	0.88	0.97	0.04	0.16
χ_*bb*_/N	1.83	1.81	2.32	2.47	–0.55	–0.54	–1.08	–1.11
χ_*cc*_/N	–4.30	–4.52	–3.46	–3.71	–0.33	–0.43	1.04	0.95

a From ref (44).

b PCS equilibrium geometries, rDSD/3F12 properties, and B3/SVP vibrational corrections from Table 1.

## Conclusions

A general computational workflow aimed at the accurate description of the conformational landscape of flexible biomolecule building blocks has been applied to α-amino acids showing peculiar features such as tautomerism, ring puckering, or different aromatic rings. Accurate structures and relative energies are obtained by the new PCS model, which combines modern double-hybrid functionals and composite wave-function methods. In particular, geometries and spectroscopic parameters are obtained at the rDSD/3F12 level, whereas improved relative energies are computed by the PCS wave-function composite method. The agreement between computed and experimental results for histidine, tryptophan, and proline permits the unbiased interpretation of the latter in terms of well-defined stereoelectronic effects, with the synergism between intra-backbone and backbone–(side chain) non-covalent interactions playing a central role.

The above results, together with those of refs (39) and (42), provide a general panorama of natural α-amino acids. In more general terms, the reasonable cost and black-box implementation of the PCS model pave the way toward accurate studies of flexible prebiotic molecules containing a few dozen atoms also by nonspecialists.

## Supporting Information Available

Different contributions to the relative energies and Cartesian coordinates of the PCS equilibrium geometries for all the low-energy structures discussed in the main text (PDF)
