# Supplementary material for: DFT Meets Wave-Function Methods for Accurate Structures and Rotational Constants of Histidine, Tryptophan, and Proline
Source: J Phys Chem A. 2023 Sep 4;127(36):7534–43. doi: 10.1021/acs.jpca.3c04227 (PMC10510395; doi:10.1021/acs.jpca.3c04227)
Supplement: Supplementary file 1 — jp3c04227_si_001.pdf [file jp3c04227_si_001.pdf]

Supporting Information: DFT Meets Wave-Function Methods for Accurate Structures and Rotational Constants of Hystidine, Tryptophan, and Proline

Vincenzo Barone,*,† Lina Marcela Uribe Grajales,†,‡ Silvia Di Grande,†,‡ Federico Lazzari,† and Marco Mendolicchio†

†*Scuola Normale Superiore di Pisa, Piazza dei Cavalieri 7, 56126 Pisa, Italy*

‡*Scuola Superiore Meridionale, Largo San Marcellino 10, 80138 Napoli, Italy*

E-mail: vincenzo.barone@sns.it

PCS energy contibutions

Table S1: PCS relative electronic energies (ΔE_{PCS}), and single contributions of the composite scheme for the low-lying tautomers and conformers of histidine. All the energetic quantities are in cm^{-1}.

Label	$\Delta(E_{V2})$	$\Delta(\Delta E_V)$	$\Delta(\Delta E_{CV2})$	ΔE_{PCS}
ϵIIgg$^-$	0	0	0	0
ϵIIg$^-$g	551	-142	5	413
δIIgg	208	-58	8	159
δIgg$^-$	1083	-100	12	995

Table S2: PCS relative electronic energies (ΔE_{PCS}), and single contributions of the composite scheme for the low-energy conformers of tryptophan. All the energetic quantities are in cm^{-1}.

Label	$\Delta(E_{V2})$	$\Delta(\Delta E_V)$	$\Delta(\Delta E_{CV2})$	ΔE_{PCS}
IIgg	0	0	0	0
IIg$^-$g	474	-85	1	390

Table S3: PCS relative electronic energies (ΔE_{PCS}), and single contributions of the composite scheme for the low-energy structures of proline. All the energetic quantities are in cm^{-1}.

Label	$\Delta(E_{V2})$	$\Delta(\Delta E_V)$	$\Delta(\Delta E_{CV2})$	ΔE_{PCS}
IIE$^-$	0	0	0	0
IIE$^+$	255	-63	9	200
IE$^-$	665	-114	10	561
IE$^+$	704	-132	7	580

PCS geometries

Histidine ϵIIgg^{-}

20

C	-2.590528	0.509162	-0.689851
N	-2.618237	-0.823035	-0.441089
C	-1.583809	-1.105866	0.417256
C	-0.970638	0.092160	0.660304
N	-1.609618	1.096040	-0.043411
C	0.247742	0.365436	1.472205
C	1.469644	0.626141	0.583215
N	1.320591	1.757845	-0.332998
C	1.799955	-0.645301	-0.210859
O	1.838855	-1.738370	0.292673
O	2.054926	-0.426257	-1.499334
H	0.335177	1.957388	-0.485400
H	2.337903	0.800871	1.223379
H	0.463394	-0.489419	2.111794
H	0.081714	1.238879	2.105893
H	-1.363290	-2.103409	0.753701
H	-3.300413	0.995147	-1.338443
H	-3.273452	-1.484081	-0.818887
H	1.930280	0.544103	-1.605722
H	1.755089	2.591191	0.035834

Histidine ϵIIg^{-}g

20

N	3.369336	-0.508812	-0.229875
C	2.211823	-1.226480	-0.042280
C	1.257541	-0.305636	0.294032
N	1.816189	0.957525	0.312282
C	3.079025	0.796365	-0.006555
C	-0.183228	-0.526142	0.599448
C	-1.118129	0.107893	-0.437359
C	-2.543768	-0.339507	-0.108291
O	-3.293374	0.613284	0.449775
N	-1.029409	1.565942	-0.384560
O	-2.937399	-1.459503	-0.301660
H	-0.093207	1.852216	-0.113593
H	-0.876850	-0.306417	-1.420259
H	-0.419455	-0.102577	1.580185
H	-0.394906	-1.594562	0.638851
H	2.170993	-2.295357	-0.157086
H	3.816146	1.578302	-0.085503
H	4.268355	-0.883493	-0.475637
H	-2.712817	1.405860	0.458882
H	-1.224478	1.974237	-1.289648

Histidine δIIgg

20

C	-1.042671	0.329821	0.540601
C	0.151716	0.816656	1.281403
C	1.418615	0.811196	0.416656
C	1.800805	-0.621390	0.027148
O	2.381116	-0.723245	-1.163222
N	1.348643	1.627689	-0.791304
O	1.609105	-1.573363	0.743137
H	0.406140	1.606133	-1.170090
H	2.255941	1.174646	1.019488
H	0.331674	0.206758	2.167802
H	-0.036133	1.838717	1.613973
H	2.329446	0.178224	-1.546685
H	1.581178	2.592356	-0.600905
N	-1.252544	-0.998382	0.247732
H	-0.624451	-1.746838	0.497905
N	-2.949477	0.096252	-0.655633
C	-2.104587	0.983542	-0.031094
H	-2.315610	2.040991	-0.005603
C	-2.404011	-1.082408	-0.467644
H	-2.795507	-2.021541	-0.823190

Histidine δIgg$^-$

20

N	-2.892006	-0.642649	-0.364471
C	-1.955082	-0.938240	0.597667
C	-0.981961	0.025795	0.641663
N	-1.343974	0.936137	-0.322706
C	-2.486387	0.485242	-0.900737
C	0.232723	0.185547	1.487036
C	1.498987	0.492246	0.675411
C	1.721598	-0.537593	-0.410567
O	1.935201	-0.282668	-1.567616
N	1.405828	1.809498	0.054812
O	1.691539	-1.787345	0.078293
H	2.000116	1.844614	-0.765043
H	2.362234	0.399568	1.347912
H	0.378863	-0.729555	2.060150
H	0.104532	1.001312	2.205385
H	-2.031160	-1.824303	1.207096
H	-2.975381	1.011519	-1.704210
H	1.709094	2.526483	0.700015
H	-0.760370	1.716791	-0.583991
H	1.829965	-2.385213	-0.669023

Tryptophan IIgg

27

C	-3.187082	0.887876	0.630019
C	-1.886343	0.984274	0.138079
C	-1.207933	-0.120408	-0.427876
C	-1.869450	-1.354790	-0.503683
C	-3.158953	-1.455008	-0.016210
C	-3.810695	-0.343837	0.546870
C	0.095040	0.336789	-0.824586
C	0.155171	1.665076	-0.491234
N	-1.031818	2.055464	0.082800
C	1.194186	-0.485298	-1.416094
C	2.148401	-1.020205	-0.342398
C	2.934318	0.141041	0.281171
O	3.120103	0.028159	1.596123
N	1.513806	-1.800753	0.719866
O	3.368559	1.060476	-0.362822
H	1.458454	-2.780188	0.479148
H	0.568656	-1.470122	0.884772
H	2.903621	-1.651708	-0.819330
H	1.777574	0.106150	-2.121439
H	0.772974	-1.331851	-1.963547
H	0.969066	2.361065	-0.616490
H	-1.234269	2.980335	0.415123
H	-1.382130	-2.217159	-0.943771
H	-3.681024	-2.401640	-0.070255
H	-4.820624	-0.454313	0.919980
H	-3.692920	1.743060	1.061173
H	2.637436	-0.788751	1.845221

Tryptophan IIg^{-}g

27

```
 C  -3.674283  0.132224  0.302992
 C  -2.432283  0.673557 -0.025896
 C  -1.297865 -0.132424 -0.266928
 C  -1.420347 -1.525787 -0.180468
 C  -2.649444 -2.066916  0.145365
 C  -3.764267 -1.245117  0.386391
 C  -0.208155  0.749516 -0.584020
 C  -0.711788  2.021830 -0.521535
 N  -2.046514  1.982205 -0.185303
 C   1.198927  0.355259 -0.874683
 C   1.915129 -0.193346  0.370305
 C   3.321713 -0.609482 -0.050440
 O   4.277073  0.258686  0.299912
 N   1.982881  0.838920  1.401558
 O   3.544431 -1.608429 -0.680380
 H   2.025961  0.429849  2.326411
 H   1.147858  1.413000  1.367482
 H   1.399379 -1.097922  0.700912
 H   1.758324  1.218245 -1.246853
 H   1.238327 -0.416565 -1.645608
 H  -0.218620  2.962764 -0.709255
 H  -2.648628  2.780722 -0.107594
 H  -0.570980 -2.169607 -0.374406
 H  -2.761180 -3.141259  0.213737
 H  -4.713377 -1.700402  0.638801
 H  -4.536541  0.761884  0.483856
 H   3.813940  0.947604  0.818892
```

Proline IE$^-$

```
17

N -0.838223 -0.992738 -0.730625
C  0.092177  0.134225 -0.730352
C -0.647942  1.313756 -0.053229
C -1.674768  0.609671  0.827756
C -2.074092 -0.565169 -0.058412
C  1.355882 -0.205379  0.024587
O  1.516347 -1.172522  0.726096
O  2.315191  0.720562 -0.168565
H  0.394088  0.410520 -1.743194
H  3.080139  0.441537  0.352686
H -1.153043  1.902322 -0.820667
H  0.028645  1.976217  0.484432
H -2.510691  1.253699  1.101613
H -1.203747  0.244853  1.743628
H -2.802728 -0.238464 -0.806202
H -2.512043 -1.400065  0.487825
H -0.412902 -1.748399 -0.208100
```

Proline IE$^+$

```
17

C  1.860619 -0.797910 -0.292432
N  0.806584 -0.979236  0.707321
C -0.086361  0.171118  0.639458
C  0.670975  1.245555 -0.179428
C  2.097558  0.701797 -0.240659
C -1.412675 -0.156165 -0.003405
O -2.266546  0.884864  0.067836
O -1.702782 -1.203409 -0.524118
H -0.308640  0.545417  1.642570
H  0.257160  1.308797 -1.188002
H  0.591824  2.232381  0.271369
H  2.654791  1.078465 -1.097760
H  2.643394  0.955142  0.669614
H  1.543819 -1.091292 -1.302950
H  2.740960 -1.382912 -0.025806
H -3.083590  0.605513 -0.367470
H  0.288120 -1.834869  0.556240
```

Proline IIE^{-}

```
17

N   0.735512 -1.026549  0.582821
C  -0.124332  0.158726  0.764971
C   0.673251  1.340039  0.202552
C   1.583269  0.664040 -0.819162
C   1.979111 -0.616866 -0.098703
C  -1.444224 -0.019437  0.006074
O  -2.291849  0.832725 -0.027383
O  -1.561495 -1.197322 -0.608194
H   0.920143 -1.496187  1.456798
H  -0.392137  0.313127  1.811052
H  -0.713410 -1.653421 -0.402859
H   1.273381  1.790396  0.995541
H   0.020453  2.104896 -0.212106
H   2.444344  1.269029 -1.098489
H   1.023265  0.427311 -1.726737
H   2.778762 -0.409896  0.620301
H   2.320928 -1.401650 -0.773033
```

Proline IIE^{+}

```
17

C   1.815576 -0.814845 -0.333016
N   0.635309 -1.059677  0.515745
C  -0.116545  0.208455  0.609887
C   0.705771  1.266660 -0.155757
C   2.111039  0.672262 -0.180184
C  -1.516261  0.063759  0.022109
O  -1.787096 -1.159450 -0.439879
O  -2.296182  0.977214 -0.028970
H   0.928749 -1.357876  1.434912
H  -0.244962  0.502270  1.652702
H   0.321614  1.370143 -1.172166
H   0.645347  2.244126  0.316840
H   2.728441  1.073434 -0.983911
H   2.622043  0.854687  0.768628
H   1.561397 -1.041777 -1.371546
H   2.633839 -1.470200 -0.038269
H  -0.974883 -1.676920 -0.244848
```
